# New Health Provisions

**Published:** 1920-04-03

**Authors:** 


### New Health Provisions.

THE Parliamentary Secretary to the Minister of Health (Sir Kingsley Wood) has foreshadowed important new health legislation. Speaking at the annual conference of the Faculty of Insurance, he said hospital provision was notoriously inadequate and its financial sources precarious. There were great gaps in hospital provision. Without any blow at existing voluntary hospitals, more institutions must be provided where they were so sorely needed. There must also be established centres and clinics in the nature of preventive institutions, in order to diminish the number of those who now go straight to the final hospitals. Legislation could be anticipated which would deal with the hospital problem as part of the general health problem giving the Minister of Health power, especially, to link existing institutions with the local health services and to make further and better general provision, especially for women and children.

Dr. Addison had come to a great decision in deciding to deal with and treat tuberculosis as a whole, and not to attempt to tinker with it further under National Insurance.

A bigger fight, continued Sir Kingsley Wood, had to be put up against venereal disease. One-half of the lunacy and blindness in the country, and a vast number of infant deaths, were due to this fatal scourge. The mistaken policy of secrecy, both as to the effects and remedies of the disease, had been largely broken down mainly owing to the efforts of the Press.

It was the intention of the Minister to introduce legislation at an early date unifying and conferring proper health powers on suitable local authorities and reforming the Poor Law. It would be, no doubt, possible and advisable for the services and experience of the Poor-Law guardians to be fully utilised in connection with a new local health system more in keeping with the spirit of the time. We wanted no health reform that would justify another great upheaval in the medical profession, and whatever was done would be done in close conjunction with the profession.

